# Calling for sustainable mechanisms in moisture-based hydrovoltaic devices

**DOI:** 10.1093/nsr/nwaf315

**Published:** 2025-08-04

**Authors:** Jun Yao, Yuhang Song

**Affiliations:** Department of Electrical and Computer Engineering, University of Massachusetts, USA; Institute for Applied Life Sciences, University of Massachusetts, USA; Department of Biomedical Engineering, University of Massachusetts, USA; Department of Electrical and Computer Engineering, University of Massachusetts, USA

## Abstract

Free Electricity from Thin Air-Is It Really Possible Anywhere, Anytime?

A lightning strike cutting across the sky is a good reminder that atmospheric moisture stores vast amounts of electricity. Moisture or water vapor in the air may be considered as a secondary form of solar energy, since about half of the adsorbed solar energy eventually dissipates through water evaporation. While not all the latent energy is harnessable, a part of it in the kinetic or electrostatic form, which is still substantial, may be used for energy conversion. Compared to other natural resources such as sunlight, wind, and geothermal heat, water vapor is more accessible and less constrained by time or location. This attraction is likely one of the reasons why the field has rapidly expanded in less than a decade (Fig. 1a). A variety of terms such as moist-electric generator [1], humidity gradient-based generator [2], and Air-gen [3], have been used to describe devices capable of generating electricity through their interaction with water vapor. The collective term ‘hydrovoltaics’ [4] embodies both hope and ambition—that the field may follow a path similar to that of photovoltaics, achieving technological maturity and societal impact.

**Figure 1.**
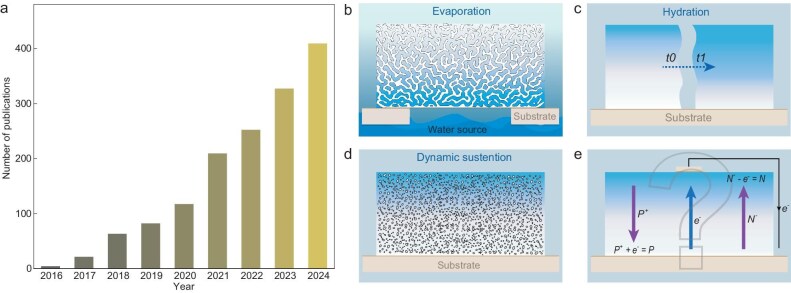
(a) Trend in publications related to energy harvesting from water vapor. The actual publication count may vary due to the broad terminology used in literature. Data is based on searches from Web of Science. (b–d) Representative strategies to create adsorption gradients in materials by (b) applying an external humidity gradient, (c) harnessing the (temporary) hydration process, and (d) spontaneous sustention through dynamic gas interaction with nanoporous structures. (e) The unknown charging mechanism for current generation. *P, N*, and *e* denote positive ion, negative ion, and electron, respectively.

However, caution is warranted. The energy density of hydrovoltaic devices typically reaches up to the order of mW/cm^3^, which is significantly lower than that of conventional batteries. Therefore, the primary appeal of hydrovoltaic technology as a more accessible energy source or for sustaining electronics [5] lies in its ability to continuously harvest energy. Photovoltaic technology is underpinned by a well-understood and fundamentally sustainable mechanism, characterized by long-term energy generation without irreversible material consumption. Likewise, to fully justify the term ‘hydrovoltaics’ and the grand promise, it is essential to identify a truly sustainable working mechanism in these devices. Without such a foundation, a hydrovoltaic device risks falling short of practical relevance, because it may not even rival a conventional chemical battery as the energy density carried by water vapor is expectedly low. However, many current studies focus more on phenomenological performance metrics than on mechanistic understandings. And the pursuit of these metrics sometimes can introduce mixed elements that further obscure insights. We intend to briefly discuss two critical aspects needed for unveiling truly sustainable mechanisms in hydrovoltaic devices.

First, like any other energy device, the generation of current flow requires a chemical potential, typically manifested in the form of a certain sustained gradient. In this regard, one category of devices meets this basic requirement by utilizing humidity gradient produced by environmental changes or water evaporation (Fig. 1b) [6]. However, reliance on environmental humidity gradients limits device deployment to areas near vapor sources such as water surfaces, nasal cavities, or human skin. For broader applicability, harnessing ambient moisture is far more attractive, but the ambient environment lacks an inherent gradient. One strategy is to use dehydrated materials to induce an adsorption gradient during hydration (Fig. 1c), but the gradient vanishes once adsorption reaches saturation [7]. Another approach is to engineer structural gradients (e.g. surface functional groups) within the device, where differential water-surface interactions mimic an adsorption gradient [8]; still, the long-term stability of surface functionality can be limited. The recent findings of sustained adsorption gradients forming spontaneously in non-gradient ambient environments in various nanoporous materials offer a more promising direction (Fig. 1d) [3,9]. It is also termed as the ‘Air-gen’ effect to highlight its spontaneous and sustained operation in ambient air. This seemingly counterintuitive effect is offered an explanation using gas behaviors in confined nanopore spaces (e.g. sizes smaller than the mean free path of water vapor), where the increased energy by gas-solid interaction is offset by a decreased gas density for maintaining a thermodynamic equilibrium with a connected open environment [3]. As a result, the increasing confinement across the thickness of a nanoporous film (along the vertical *Z*-direction) can yield a decreasing adsorption to generate a gradient, which sustains over time as it is built up from a dynamic equilibrium. This generic ‘Air-gen’ effect offers broad material choices for device engineering and investigations. Further studies may be still needed to elucidate the detailed interaction dynamics in order to improve the mechanistic understanding of its sustainability.

Second, other than meeting the basic requirement of a sustained gradient, a sustainable charging mechanism is needed to maintain closed-loop current generation. For instance, the reversible electron-hole separation and recombination in photovoltaics enable a continuous current loop, but a redox-based charge exchange in chemical batteries causes irreversible material consumption and does not support long-term continuity. Unfortunately, the charging mechanism remains largely unanswered in the field. Many existing studies tend to propose moisture-mediated ion (e.g. H^+^) diffusion as a means for the internal current generation [1]. However, these proposed mechanisms face a critical challenge: how does the internal ionic current convert to an external electron current? If this conversion involves redox processes (purple arrows, Fig. 1e), then the system begins to resemble a chemical battery, inherently limiting its sustainability. At present, this critical question remains largely unaddressed in the field. On the contrary, many studies employ active metals that are prone to oxidize in moist environments as electrodes. Although these electrodes can often enhance energy output, they likely contribute to current production via redox reactions. If the component material is consumed over time, the device effectively functions as a moisture-mediated chemical battery [10] and loses its competitiveness against conventional batteries. It is important to note that open-circuit voltage measurements, commonly used to evaluate performance, are not sufficient to demonstrate sustainable energy generation. Voltage measurement does not involve energy extraction from the device, and a chemical battery can maintain open-circuit voltage over extended periods. Current measurements offer a more direct indication of energy generation, but they are often reported over shorter time spans in many studies—subtly indicating the challenges of sustaining electricity generation. A practical reference for defining ‘long-term’ performance in current measurements is to ensure that the cumulative charge or energy output exceeds the amount that could be potentially released from stored chemical energy within the materials. So far, it is fair to say that the demonstrated current productions have not matched the continuity of typical chemical batteries. Still, these phenomenological performances alone cannot confirm or refute the existence of truly sustainable mechanisms. For instance, a recent study offers support to a sustainable mechanism by viewing the interaction between water molecules and a solid interface as a form of gas-solid ‘contact electrification’ [3], based on the understanding that water adsorption on a solid interface is a dynamic exchange process (the estimated charge transfer rate is on the order of µC/cm²‧s [9]). As a result, the spontaneously sustained adsorption gradient (previously discussed) is expected to generate an electrification gradient (e.g. the high-adsorption region is ‘rubbed’ by more water molecules than the low-adoption region). This differentiated ‘contact electrification’ could, in principle, sustain continuous device charging, assuming that open air can provide an unlimited supply of fresh water molecules. The fact that this is a generic effect found in almost all materials further supports the possibility [3]. If an electron-transfer mechanism at the water-solid interface similar to that proposed in triboelectricity (triboelectricity is similar to contact electrification but can involve sliding) is assumed, it could, in theory, sustain a closed-loop current flow. An alternative but similar view is to consider ‘image electrons’ induced in the material by water-assisted surface ionizations [6,11]. These proposed mechanisms lead to an internal electron current flow consistent with the external one (blue arrow, Fig. 1e), sustaining current generation without the need of a redox process. However, the exact charge-transfer process remains unclear and warrants further investigation. Uncovering the detailed charging mechanism may require new approaches, since surface charging processes (e.g. those involved in streaming currents and triboelectricity) are inherently complex and challenging to probe. Advanced techniques such as ultrafast spectroscopies, which have so far seen limited use in this field, could offer valuable insights into the underlying charge transfer dynamics.

In summary, the overarching promise of hydrovoltaic technology hinges on unveiling a truly sustainable mechanism. While the relatively simple structure and characterization of hydrovoltaic devices lower the entry barrier to the field, they may also promote an overflow with narrow focus on certain phenomenological metrics. Such a ‘burgeoning’ trend can be misleading or even delay long-term development, by clouding key understandings and diverting attention away from the critical areas that need focus. Greater synergistic efforts are needed to deepen the fundamental understanding of charging and transport mechanisms, in order to justify, accelerate, and advance technological development in hydrovoltaics.

## References

[1] Shen D, Duley WW, Peng P et al. Adv Mater 2020; 32: 2003722. 10.1002/adma.202003722 33185944

[2] Yan H, Liu Z, Qi R. Nano Energy 2022; 101: 107591. 10.1016/j.nanoen.2022.107591

[3] Liu X, Gao H, Sun L et al. Adv Mater 2024; 36: 2300748. 10.1002/adma.202300748 37144425

[4] Zhang Z, Li X, Yin J et al. Nat Nanotechnol 2018; 13: 1109–19. 10.1038/s41565-018-0228-6 30523296

[5] Fu T, Liu X, Fu S et al. Nat Commun 2021; 12: 3351. 10.1038/s41467-021-23744-2 34099691 PMC8184933

[6] Liu X, Ueki T, Gao H et al. Nat Commun 2022; 13: 4369. 10.1038/s41467-022-32105-6 35902587 PMC9334603

[7] Bai J, Huang Y, Wang H et al. Adv Mater 2022; 34: 2103897. 10.1002/adma.202103897 34965320

[8] Cheng H, Huang Y, Zhao F et al. Energy Environ Sci 2018; 11: 2839. 10.1039/C8EE01502C

[9] Liu X, Gao H, Ward JE et al. Nature 2020; 578: 550–4. 10.1038/s41586-020-2010-9 32066937

[10] Zhang Y, Nandakumar DK, Tan SC. Joule 2020; 4: 2532–6. 10.1016/j.joule.2020.10.003

[11] Qing Y, Wang Y, Sun X et al. Angew Chem Int Ed 2020; 59: 10619–25. 10.1002/anie.202002762 32187779

